# Community Intelligence in Knowledge Curation: An Application to Managing Scientific Nomenclature

**DOI:** 10.1371/journal.pone.0056961

**Published:** 2013-02-25

**Authors:** Lin Dai, Chao Xu, Ming Tian, Jian Sang, Dong Zou, Ang Li, Guocheng Liu, Fei Chen, Jiayan Wu, Jingfa Xiao, Xumin Wang, Jun Yu, Zhang Zhang

**Affiliations:** 1 School of Computer Science and Technology, Beijing Institute of Technology, Beijing, China; 2 CAS Key Laboratory of Genome Sciences and Information, Beijing Institute of Genomics, Chinese Academy of Sciences, Beijing, China; 3 Research Institute of Subtropical Forestry, Chinese Academy of Forestry, Fuyang, Zhejiang, China; University of Lausanne, Switzerland

## Abstract

Harnessing community intelligence in knowledge curation bears significant promise in dealing with communication and education in the flood of scientific knowledge. As knowledge is accumulated at ever-faster rates, scientific nomenclature, a particular kind of knowledge, is concurrently generated in all kinds of fields. Since nomenclature is a system of terms used to name things in a particular discipline, accurate translation of scientific nomenclature in different languages is of critical importance, not only for communications and collaborations with English-speaking people, but also for knowledge dissemination among people in the non-English-speaking world, particularly young students and researchers. However, it lacks of accuracy and standardization when translating scientific nomenclature from English to other languages, especially for those languages that do not belong to the same language family as English. To address this issue, here we propose for the first time the application of community intelligence in scientific nomenclature management, namely, harnessing collective intelligence for translation of scientific nomenclature from English to other languages. As community intelligence applied to knowledge curation is primarily aided by wiki and Chinese is the native language for about one-fifth of the world’s population, we put the proposed application into practice, by developing a wiki-based English-to-Chinese Scientific Nomenclature Dictionary (ESND; http://esnd.big.ac.cn). ESND is a wiki-based, publicly editable and open-content platform, exploiting the whole power of the scientific community in collectively and collaboratively managing scientific nomenclature. Based on community curation, ESND is capable of achieving accurate, standard, and comprehensive scientific nomenclature, demonstrating a valuable application of community intelligence in knowledge curation.

## Introduction

With the exponentially increasing volume of knowledge, harnessing community intelligence in knowledge curation has gained significant attention to deal with communication and education in the flood of scientific knowledge [1], [2]. A successful example that engages community intelligence in knowledge aggregation is Wikipedia (http://www.wikipedia.org), an online encyclopedia allowing any user to create/edit any content. Although the openness of editorial capacity to the community may lead to potential vandalism, it is reported that Wikipedia not only achieves more content coverage than BBC (British Broadcasting Corporation) and CNN (Cable News Network) combined [3] but also rivals the traditional Encyclopedia in accuracy [4], [5]. Spirited by the extraordinary success of Wikipedia, it has been advocated, for instance, in life sciences, that biological knowledge databases go wiki [6]. Meanwhile, leading voices in biological knowledge curation (e.g., gene annotation in model organism) published an article in *Nature* to elaborate the current state and future of knowledge curation; they stated that keeping biological knowledge up-to-date and comprehensive is increasingly lagging behind knowledge creation, inevitably requiring a large number of people getting involved in knowledge curation [7]. In other words, community curation is critical for biological knowledge management due to the burgeoning volume of biological knowledge and insufficient funding for dedicated curators [8]. As wiki features community-based knowledge curation, up-to-date content, and low cost for maintenance [9] – although there are limitations of using open wikis for knowledge management [10], more than a dozen biological knowledge wikis have been constructed to date [11]–[29], attempting to exploit the whole power of the scientific community for collective and collaborative knowledge curation [30], [31].

As knowledge is accumulated at ever-faster rates, a large collection of scientific nomenclature is concurrently developed or evolved in all kinds of fields. Scientific nomenclature, a particular kind of knowledge, is a system of terms used to name things and to describe processes/phenomenon in a particular discipline. Rapid advancements in science and technology further lead to a growing quantity of new scientific terms. Therefore, accurate translation of scientific nomenclature in different languages is of critical significance, not only for effectively facilitating communications and collaborations with English-speaking people, but also for efficiently disseminating knowledge among people in the non-English-speaking world, especially young students and researchers, and thus laying a solid foundation for domestic scientific activities. An accurate translation of a scientific term should be not only expressed equivalently in another language but also widely accepted by the scientific community. However, there are inaccurate and/or unacknowledged translations of scientific nomenclature from English to other languages, particularly for those languages that do not belong to the same language family as English [32]. Taking Chinese as an example, due to differences in language and culture between China and western countries, the English-to-Chinese scientific nomenclature lacks of accuracy and standardization [33]–[36], which can severely affect knowledge exchange and hinder the scientific activities.

To address this issue, here we propose for the first time the application of community intelligence in scientific nomenclature management, namely, harnessing collective intelligence for translation of scientific nomenclature from English to other languages. As community intelligence applied to knowledge curation is primarily aided by wiki and Chinese is the native language for about one-fifth of the world’s population (or over one billion people) [37], we put the proposed application into practice by developing a wiki-based English-to-Chinese Scientific Nomenclature Dictionary (ESND). ESND is a wiki-based, publicly editable and open-content platform, aiming to exploit the whole power of the scientific community in building standard and accurate scientific nomenclature.

## Methods

ESND is built on wiki, which was first proposed by Ward Cunningham [38]. Briefly speaking, a wiki is an open website that allows users to add, modify, or delete its content via a web browser using a simplified markup language or a rich-text editor. As illustrated by Wikipedia and other wiki-based projects [39]–[41], wiki has several key features. First, it allows any user to create new pages or to edit any page (with customized permission control for editing), using the web browser without any extra add-ones. Second, it enables web contents to be written collectively and collaboratively by multiple users. Third, it builds a quite simple website, facilitating users to be involved in an ongoing process of creation and collaboration that constantly changes the website layout. Thus, wiki can significantly ease the process of knowledge creation, curation, and sharing. The wide adoption of community intelligence in knowledge management is to some extent attributable to free wiki software in aid of its implementation, such as MediaWiki (http://www.mediawiki.org), a free, open-source, and widely used wiki engine (e.g., adopted by Wikipedia). To develop ESND, we used MediaWiki (Version 1.19.1), MySQL (http://www.mysql.org; a free and popular relational database management system, Version 5.1.58), and PHP (http://www.php.net; a widely-used general-purpose scripting language, Version 5.2.17) on a Red Hat Enterprise Linux Server.

## Results and Discussion

### Community Curation of Scientific Nomenclature

ESND is a wiki-based, publicly editable, open-content platform for collaborative management of English-to-Chinese scientific nomenclature. Scientific nomenclature is a system of terms in different fields. In ESND, terms are extracted from the scientific literature and classified according to their corresponding fields. Currently, ESND primarily focuses on biological terms; it contains 2679 terms in life sciences, 770 terms in computer science and 135 terms in chemistry.

**Figure 1:**
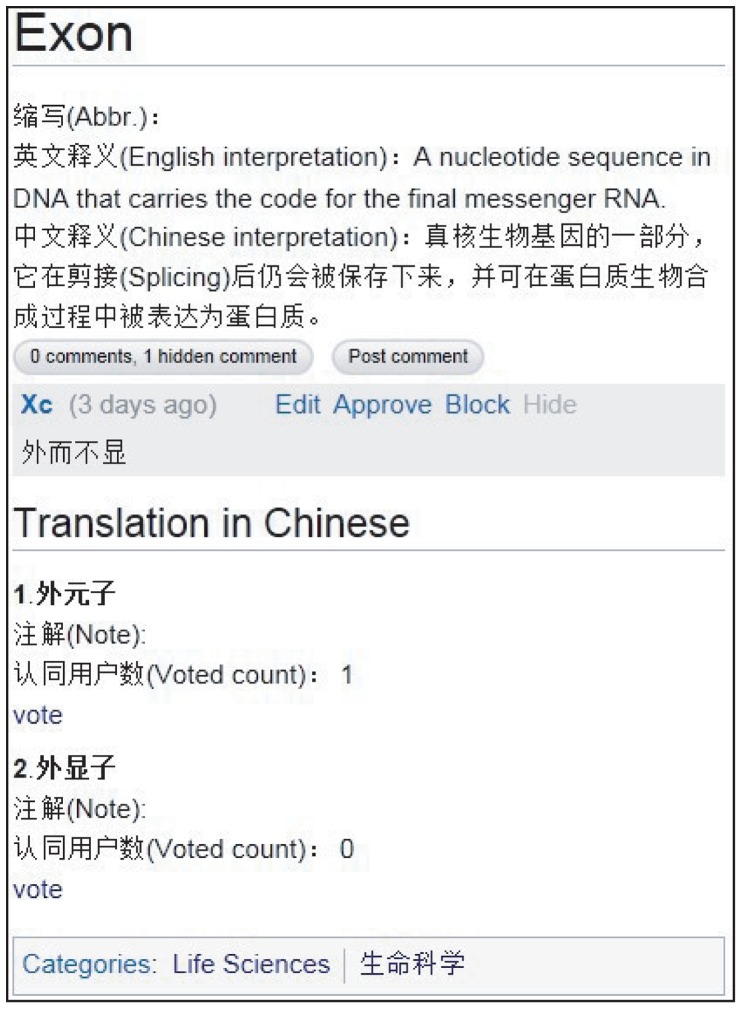
Screenshot of the term “Exon” in ESND (http://esnd.big.ac.cn/index.php/Exon).

In ESND, each wiki page represents a specific term, which generally contains a collection of relevant information, including English abbreviation, English interpretation, Chinese interpretation, Chinese translation(s) and the corresponding detailed note(s) (Figure 1). Specifically, English and Chinese interpretations are definitions expressed in the two languages. Each term may contain more than one Chinese translation (detailed below) and the note provides a detailed explanation for the corresponding translation.

It is likely that one English term may have multiple Chinese translations. In life sciences, for instance, there are two terms “exon” and “intron” that are often translated as “
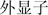
” (Pinyin: *wài xiǎn zǐ* and “
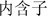
” (Pinyin: *nèi hán zǐ*). However, recent studies have demonstrated that not all exons are encoded into proteins and not all introns are not transcribed, advocating that “
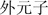
” (Pinyin: *wài yuán zǐ*) and “
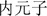
” (Pinyin: *nèi yuán zǐ*) are more appropriate translations, respectively [42], [43]. Another example is “evolution”, which also has multiple translations: “
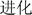
” (Pinyin: *jìn huà*) and “
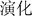
” (*yǎn huà*). Nowadays, some people believe that it might be better translated as “
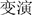
” (Pinyin: *biàn yǎn*), since “
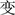
” (Pinyin: *biàn*) indicates mutation and “
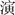
” (Pinyin: *yǎn*) represents the process of natural selection.

To tackle this issue, ESND incorporates a “vote” function for each wiki page to engage community intelligence in dealing with multiple translations for a given English term. The number of votes indicates the recognition of the corresponding translation accepted by the scientific community. For the term “exon” as mentioned above, higher vote for a Chinese translation indicates that it is more acknowledged by the scientific community (Figure 1). Therefore, community-based votes effectively solve this issue by harnessing community intelligence in standardizing English-to-Chinese nomenclature. In addition, every term in ESND incorporates an open comment function as well as a corresponding discussion page, which can help users make comment and raise discussion for any debatable issue.

**Figure 2:**
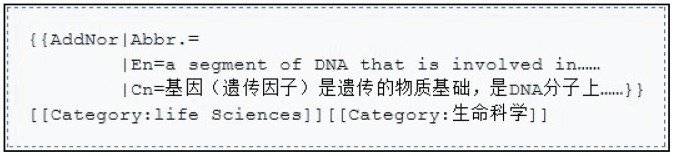
Example for adding a term to ESND.

To facilitate adding new terms, ESND provides an open template for creating new wiki pages in correspondence to new terms. Users can adopt the template (http://esnd.big.ac.cn/index.php/template:AddNor) to easily add a new term. For example, when adding the term “Gene” (Figure 2), “Abbr” is the abbreviation of the term if available, “En” is its English interpretation, “Cn” is its Chinese interpretation, and “Category” is the field that the term belongs to.

ESND is freely available at http://esnd.big.ac.cn, allowing any user to view and search for any content. Most important, ESND is a collaborative platform for collective management of English-to-Chinese scientific nomenclature, allowing people to participate in sharing their knowledge on specific terms of their own interest. If one term is not available, or incomplete, or you think of a better translation, you can share your knowledge by adding information to ESND. In addition, any user can make vote when there are multiple translations available for a given English term and thus the most voted translation indicates the wide acceptance by the scientific community. “*With enough eyeballs, all bugs are shallow*” [44]. Aided by wiki technology, ESND is a community-based dynamic resource of English-to-Chinese scientific nomenclature.

### Future Developments and Perspective

Future developments include the addition of more scientific nomenclature from different fields and the improvement of user interface for term management. To our knowledge, it is for the first time that collective intelligence is applied for community management of scientific nomenclature and ESND is the first implementation to harnessing community intelligence for scientific nomenclature management. Based on community curation, ESND is capable of achieving accurate, standard, and comprehensive scientific nomenclature, demonstrating a valuable application of community intelligence in knowledge curation.
